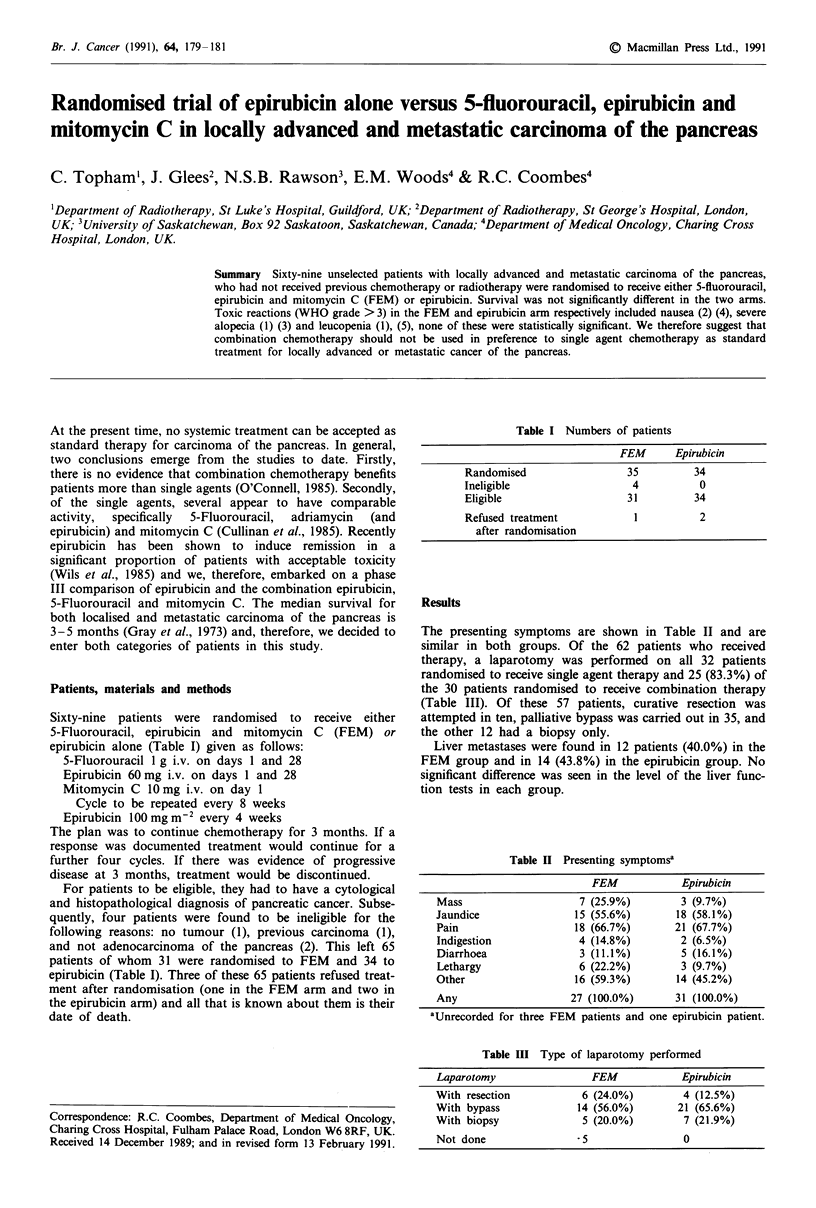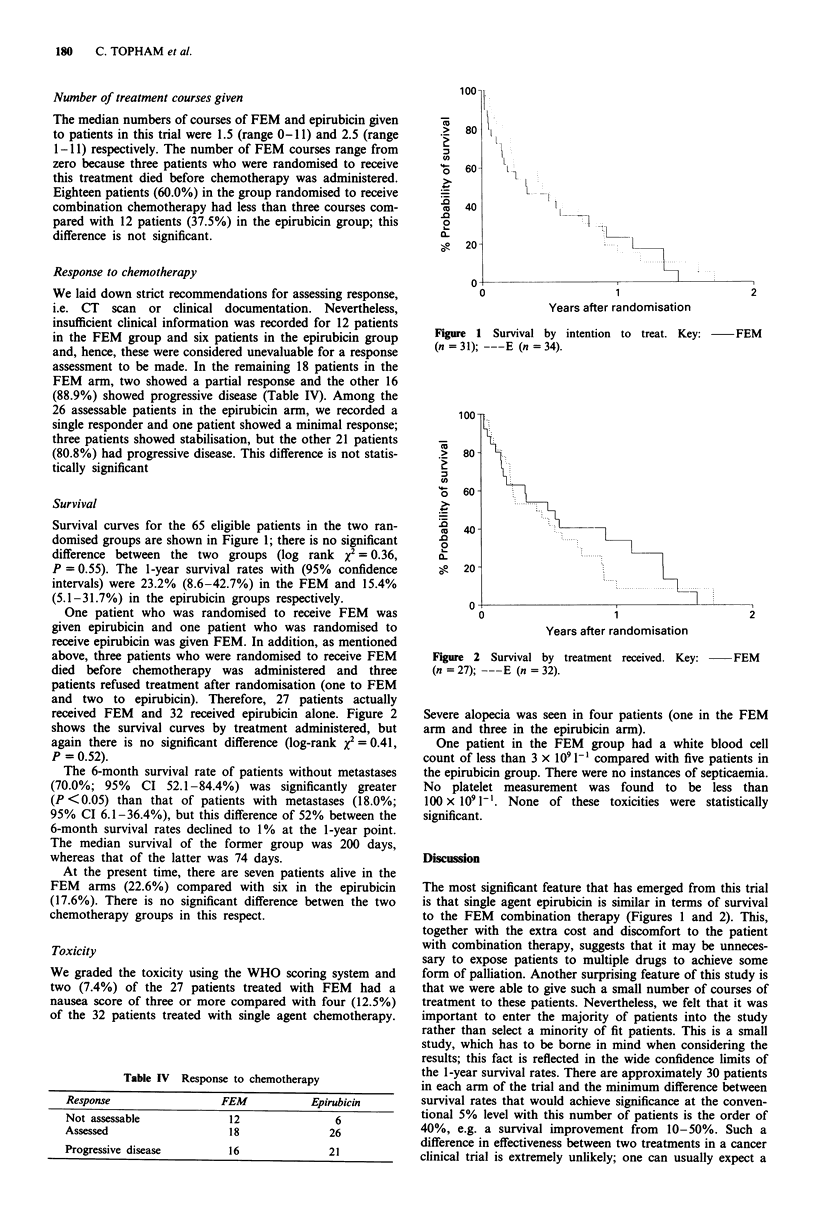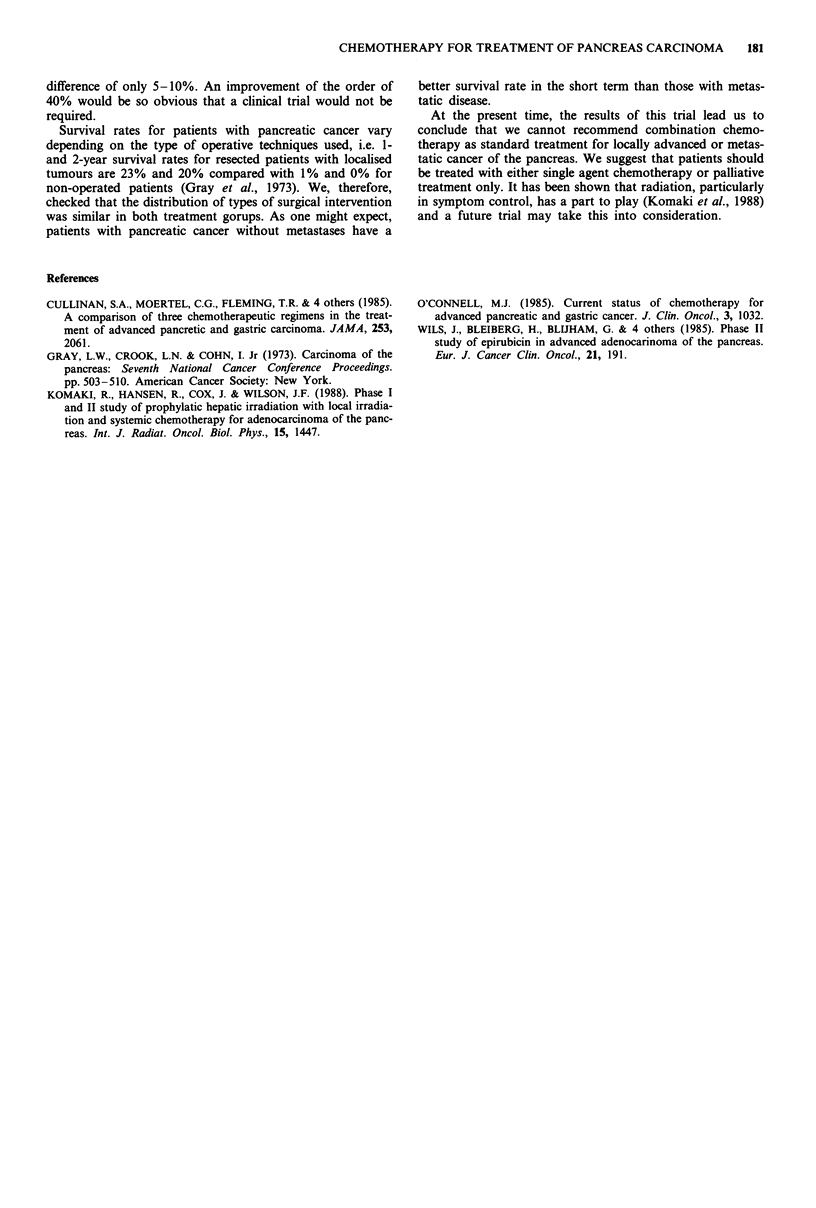# Randomised trial of epirubicin alone versus 5-fluorouracil, epirubicin and mitomycin C in locally advanced and metastatic carcinoma of the pancreas.

**DOI:** 10.1038/bjc.1991.265

**Published:** 1991-07

**Authors:** C. Topham, J. Glees, N. S. Rawson, E. M. Woods, R. C. Coombes

**Affiliations:** Department of Radiotherapy, St Luke's Hospital, Guildford, UK.

## Abstract

Sixty-nine unselected patients with locally advanced and metastatic carcinoma of the pancreas, who had not received previous chemotherapy or radiotherapy were randomised to receive either 5-fluorouracil, epirubicin and mitomycin C (FEM) or epirubicin. Survival was not significantly different in the two arms. Toxic reactions (WHO grade greater than 3) in the FEM and epirubicin arm respectively included nausea (2), (4), severe alopecia (1) (3) and leucopenia (1), (5), none of these were statistically significant. We therefore suggest that combination chemotherapy should not be used in preference to single agent chemotherapy as standard treatment for locally advanced or metastatic cancer of the pancreas.


*Br. J. Cancer* (1991), **64**, 179–181 © Macmillan Press Ltd., 1991

### Randomised trial of epirubicin alone versus 5-fluorouracil, epirubicin and mitomycin C in locally advanced and metastatic carcinoma of the pancreas

C. Topham[1], J. Glees[2], N.S.B. Rawson[3], E.M. Woods[4] & R.C. Coombes[4]

[1]*Department of Radiotherapy, St Luke's Hospital, Guildford, UK;* [2]*Department of Radiotherapy, St George's Hospital, London, UK;* [3]*University of Saskatchewan, Box 92 Saskatoon, Saskatchewan, Canada;* [4]*Department of Medical Oncology, Charing Cross Hospital, London, UK.*

**Summary** Sixty-nine unselected patients with locally advanced and metastatic carcinoma of the pancreas, who had not received previous chemotherapy or radiotherapy were randomised to receive either 5-fluorouracil, epirubicin and mitomycin C (FEM) or epirubicin. Survival was not significantly different in the two arms. Toxic reactions (WHO grade >3) in the FEM and epirubicin arm respectively included nausea (2) (4), severe alopecia (1) (3) and leucopenia (1), (5), none of these were statistically significant. We therefore suggest that combination chemotherapy should not be used in preference to single agent chemotherapy as standard treatment for locally advanced or metastatic cancer of the pancreas.

At the present time, no systemic treatment can be accepted as standard therapy for carcinoma of the pancreas. In general, two conclusions emerge from the studies to date. Firstly, there is no evidence that combination chemotherapy benefits patients more than single agents (O'Connell, 1985). Secondly, of the single agents, several appear to have comparable activity, specifically 5-Fluorouracil, adriamycin (and epirubicin) and mitomycin C (Cullinan *et al.*, 1985). Recently epirubicin has been shown to induce remission in a significant proportion of patients with acceptable toxicity (Wils *et al.*, 1985) and we, therefore, embarked on a phase III comparison of epirubicin and the combination epirubicin, 5-Fluorouracil and mitomycin C. The median survival for both localised and metastatic carcinoma of the pancreas is 3–5 months (Gray *et al.*, 1973) and, therefore, we decided to enter both categories of patients in this study.

### Patients, materials and methods

Sixty-nine patients were randomised to receive either 5-Fluorouracil, epirubicin and mitomycin C (FEM) *or* epirubicin alone (Table I) given as follows:

5-Fluorouracil 1 g i.v. on days 1 and 28
Epirubicin 60 mg i.v. on days 1 and 28
Mitomycin C 10 mg i.v. on day 1
Cycle to be repeated every 8 weeks
Epirubicin 100 mg $m^{-2}$ every 4 weeks

The plan was to continue chemotherapy for 3 months. If a response was documented treatment would continue for a further four cycles. If there was evidence of progressive disease at 3 months, treatment would be discontinued.

For patients to be eligible, they had to have a cytological and histopathological diagnosis of pancreatic cancer. Subsequently, four patients were found to be ineligible for the following reasons: no tumour (1), previous carcinoma (1), and not adenocarcinoma of the pancreas (2). This left 65 patients of whom 31 were randomised to FEM and 34 to epirubicin (Table I). Three of these 65 patients refused treatment after randomisation (one in the FEM arm and two in the epirubicin arm) and all that is known about them is their date of death.

Correspondence: R.C. Coombes, Department of Medical Oncology, Charing Cross Hospital, Fulham Palace Road, London W6 8RF, UK.
Received 14 December 1989; and in revised form 13 February 1991.

**Table I** Numbers of patients

| | *FEM* | *Epirubicin* |
|---|---|---|
| Randomised | 35 | 34 |
| Ineligible | 4 | 0 |
| Eligible | 31 | 34 |
| Refused treatment after randomisation | 1 | 2 |

### Results

The presenting symptoms are shown in Table II and are similar in both groups. Of the 62 patients who received therapy, a laparotomy was performed on all 32 patients randomised to receive single agent therapy and 25 (83.3%) of the 30 patients randomised to receive combination therapy (Table III). Of these 57 patients, curative resection was attempted in ten, palliative bypass was carried out in 35, and the other 12 had a biopsy only.

Liver metastases were found in 12 patients (40.0%) in the FEM group and in 14 (43.8%) in the epirubicin group. No significant difference was seen in the level of the liver function tests in each group.

**Table II** Presenting symptoms[a]

| | *FEM* | *Epirubicin* |
|---|---|---|
| Mass | 7 (25.9%) | 3 (9.7%) |
| Jaundice | 15 (55.6%) | 18 (58.1%) |
| Pain | 18 (66.7%) | 21 (67.7%) |
| Indigestion | 4 (14.8%) | 2 (6.5%) |
| Diarrhoea | 3 (11.1%) | 5 (16.1%) |
| Lethargy | 6 (22.2%) | 3 (9.7%) |
| Other | 16 (59.3%) | 14 (45.2%) |
| Any | 27 (100.0%) | 31 (100.0%) |

[a]Unrecorded for three FEM patients and one epirubicin patient.

**Table III** Type of laparotomy performed

| *Laparotomy* | *FEM* | *Epirubicin* |
|---|---|---|
| With resection | 6 (24.0%) | 4 (12.5%) |
| With bypass | 14 (56.0%) | 21 (65.6%) |
| With biopsy | 5 (20.0%) | 7 (21.9%) |
| Not done | ·5 | 0 |

*Number of treatment courses given*

The median numbers of courses of FEM and epirubicin given to patients in this trial were 1.5 (range 0–11) and 2.5 (range 1–11) respectively. The number of FEM courses range from zero because three patients who were randomised to receive this treatment died before chemotherapy was administered. Eighteen patients (60.0%) in the group randomised to receive combination chemotherapy had less than three courses compared with 12 patients (37.5%) in the epirubicin group; this difference is not significant.

*Response to chemotherapy*

We laid down strict recommendations for assessing response, i.e. CT scan or clinical documentation. Nevertheless, insufficient clinical information was recorded for 12 patients in the FEM group and six patients in the epirubicin group and, hence, these were considered unevaluable for a response assessment to be made. In the remaining 18 patients in the FEM arm, two showed a partial response and the other 16 (88.9%) showed progressive disease (Table IV). Among the 26 assessable patients in the epirubicin arm, we recorded a single responder and one patient showed a minimal response; three patients showed stabilisation, but the other 21 patients (80.8%) had progressive disease. This difference is not statistically significant

*Survival*

Survival curves for the 65 eligible patients in the two randomised groups are shown in Figure 1; there is no significant difference between the two groups (log rank $\chi^2 = 0.36$, $P = 0.55$). The 1-year survival rates with (95% confidence intervals) were 23.2% (8.6–42.7%) in the FEM and 15.4% (5.1–31.7%) in the epirubicin groups respectively.

One patient who was randomised to receive FEM was given epirubicin and one patient who was randomised to receive epirubicin was given FEM. In addition, as mentioned above, three patients who were randomised to receive FEM died before chemotherapy was administered and three patients refused treatment after randomisation (one to FEM and two to epirubicin). Therefore, 27 patients actually received FEM and 32 received epirubicin alone. Figure 2 shows the survival curves by treatment administered, but again there is no significant difference (log-rank $\chi^2 = 0.41$, $P = 0.52$).

The 6-month survival rate of patients without metastases (70.0%; 95% CI 52.1–84.4%) was significantly greater ($P < 0.05$) than that of patients with metastases (18.0%; 95% CI 6.1–36.4%), but this difference of 52% between the 6-month survival rates declined to 1% at the 1-year point. The median survival of the former group was 200 days, whereas that of the latter was 74 days.

At the present time, there are seven patients alive in the FEM arms (22.6%) compared with six in the epirubicin (17.6%). There is no significant difference betwen the two chemotherapy groups in this respect.

*Toxicity*

We graded the toxicity using the WHO scoring system and two (7.4%) of the 27 patients treated with FEM had a nausea score of three or more compared with four (12.5%) of the 32 patients treated with single agent chemotherapy.

**Table IV** Response to chemotherapy

| *Response* | *FEM* | *Epirubicin* |
|---|---|---|
| Not assessable | 12 | 6 |
| Assessed | 18 | 26 |
| Progressive disease | 16 | 21 |

**Figure 1** Survival by intention to treat. Key: ——FEM ($n = 31$); ---E ($n = 34$).

**Figure 2** Survival by treatment received. Key: ——FEM ($n = 27$); ---E ($n = 32$).

Severe alopecia was seen in four patients (one in the FEM arm and three in the epirubicin arm).

One patient in the FEM group had a white blood cell count of less than $3 \times 10^9 l^{-1}$ compared with five patients in the epirubicin group. There were no instances of septicaemia. No platelet measurement was found to be less than $100 \times 10^9 l^{-1}$. None of these toxicities were statistically significant.

### Discussion

The most significant feature that has emerged from this trial is that single agent epirubicin is similar in terms of survival to the FEM combination therapy (Figures 1 and 2). This, together with the extra cost and discomfort to the patient with combination therapy, suggests that it may be unnecessary to expose patients to multiple drugs to achieve some form of palliation. Another surprising feature of this study is that we were able to give such a small number of courses of treatment to these patients. Nevertheless, we felt that it was important to enter the majority of patients into the study rather than select a minority of fit patients. This is a small study, which has to be borne in mind when considering the results; this fact is reflected in the wide confidence limits of the 1-year survival rates. There are approximately 30 patients in each arm of the trial and the minimum difference between survival rates that would achieve significance at the conventional 5% level with this number of patients is the order of 40%, e.g. a survival improvement from 10–50%. Such a difference in effectiveness between two treatments in a cancer clinical trial is extremely unlikely; one can usually expect a difference of only 5–10%. An improvement of the order of 40% would be so obvious that a clinical trial would not be required.

Survival rates for patients with pancreatic cancer vary depending on the type of operative techniques used, i.e. 1- and 2-year survival rates for resected patients with localised tumours are 23% and 20% compared with 1% and 0% for non-operated patients (Gray *et al.*, 1973). We, therefore, checked that the distribution of types of surgical intervention was similar in both treatment gorups. As one might expect, patients with pancreatic cancer without metastases have a better survival rate in the short term than those with metastatic disease.

At the present time, the results of this trial lead us to conclude that we cannot recommend combination chemotherapy as standard treatment for locally advanced or metastatic cancer of the pancreas. We suggest that patients should be treated with either single agent chemotherapy or palliative treatment only. It has been shown that radiation, particularly in symptom control, has a part to play (Komaki *et al.*, 1988) and a future trial may take this into consideration.

### References

CULLINAN, S.A., MOERTEL, C.G., FLEMING, T.R. & 4 others (1985). A comparison of three chemotherapeutic regimens in the treatment of advanced pancretic and gastric carcinoma. *JAMA*, **253**, 2061.

GRAY, L.W., CROOK, L.N. & COHN, I. Jr (1973). Carcinoma of the pancreas: *Seventh National Cancer Conference Proceedings*. pp. 503–510. American Cancer Society: New York.

KOMAKI, R., HANSEN, R., COX, J. & WILSON, J.F. (1988). Phase I and II study of prophylatic hepatic irradiation with local irradiation and systemic chemotherapy for adenocarcinoma of the pancreas. *Int. J. Radiat. Oncol. Biol. Phys.*, **15**, 1447.

O'CONNELL, M.J. (1985). Current status of chemotherapy for advanced pancreatic and gastric cancer. *J. Clin. Oncol.*, **3**, 1032.

WILS, J., BLEIBERG, H., BLIJHAM, G. & 4 others (1985). Phase II study of epirubicin in advanced adenocarinoma of the pancreas. *Eur. J. Cancer Clin. Oncol.*, **21**, 191.